# Supplementary material for: Logged tropical forests have amplified and diverse ecosystem energetics
Source: Nature. 2022 Dec 14;612(7941):707–13. doi: 10.1038/s41586-022-05523-1 (PMC9771799; doi:10.1038/s41586-022-05523-1)
Supplement: Supplementary file 1 — This file contains Supplementary Discussion, References, Tables 1–3 and description of Supplementary Data. [file 41586_2022_5523_MOESM1_ESM.pdf]

nature portfolio

https://doi.org/10.1038/s41586-022-05523-1

Supplementary information

Logged tropical forests have amplified and diverse ecosystem energetics

In the format provided by the authors and unedited

Logged tropical forests have amplified and diverse ecosystem energetics

Supplementary Discussion

Shifted baselines

Could the lower energetics in the old growth forests be partially explained by past defaunation and shifted baselines?

While there might be some declines in faunal abundances in VJR due to its position as a large fragment between logged and oil palm landscapes, the Maliau, Danum and Sepilok study sites are well-protected large areas, experience very low hunting pressure and are unlikely to have experienced any substantive decline in energetically dominant fauna because of anthropogenic pressure [1]). These sites are the focus on intensive research in the SAFE project and other projects and in their literature there is no indication of the OG forests being unusually depauperate compared to other OG forests in Borneo. Indeed, many studies point out how intact and abundant the fauna appear to be because of low hunting pressure (e.g. [1]).

Logged forest recovery after logging

Could these logged forests be considered as recovered forests after degradation?

These forests have been logged a number of times, with the most recent logging in the mid-2000s [2]. As shown from the structure and biomass (Figure ED1) these are very far from structural recovery [3]

Possible effect of dietary shifts across habitat types

How much could dietary shifts within species across habitat types affect our results?

We do include a random uncertainty in our assignment of diet preferences but do not include a systematic uncertainty as there is little evidence of a shift in diet types across the land use types. As we focus on the energetic uptake through various food pathways, the only way that changes in diet affect our calculations is through variation is assimilation efficiency. The assimilation efficiency is very similar among most food types (around 70-90%; see Table S2), with the notable exception being leaves, which are less nutritious and palatable (assimilation efficiency around 30-50% in mammals, apparently higher in birds where they are a minimal part of diets). Hence the only shift in dietary type that would have a noticeable impact on our calculations would be one from leaves to other foodstuffs. There is very unlikely to be a major shift in diet from leaves to other food types because of constraints of feeding and gut morphology. There is more evidence of dietary shift within a feeding guild, *e.g.*, from feeding on herbivorous arthropods to predators (*e.g.* spiders). We directly consider this shift in trophic level of insectivorous feeding in our calculations of indirect energy consumption.

To test the sensitivity to dietary shift we ran two additional models for the mixed feeders: one with a 30% shift towards arthropods and another with a 30% shift towards leaves. We only considered species that had that arthropods or leaves as part of their diet, and increased the arthropod or leaf consumption at the expense of other food groups. The 30% shift towards arthropods results in a negligible 0.20% increase in total energy consumption. This is due to the energetic properties of all food types except leaves being very similar, and very few species in our dataset feeding on both leaves and invertebrates. However, this diet shift would lead to a bigger increase, 8.9%, in the amount of NPP required to support insectivory (assuming a mean trophic level of 2.5 for consumed invertebrates), from 51% of NPP to 55% of NPP. If we assume a 30% shift towards leaves at the expense of seeds, nuts and fruits for mixed herbivores in logged forest, this results in only a small (2.3%) increase in energy consumption compared with no shift in their diet.

<u>Geographical effects</u>

Could the proximity of some of the old growth forests to the logged forest landscape affect the results? Conversely, could the separation of some of the old growth sites result in local environmental factors (e.g., soil fertility) causing differences between the old growth and logged forest sites?

If our results were driven by strong spatial contrast between sites (*e.g.,* driven by geology or environmental factors), we expect strong differences between the local old growth site (VJR) and the more distant ones. Conversely, if local source-sink effects were affecting our proximal old growth site, we would also expect differences in energetics between local and distant old growth sites.

To examine differences between the various old growth forests we separately analysed the bird and mammal energetics for VJR, Maliau (birds and mammals) and Danum and Sepilok (birds only; Figure ED5). This shows relatively little difference in overall bird and mammal energetics between the various OG sites compared with the logged forests, and the loggest forest energetics are consistently higher. This strongly suggests that both geographical and proximity effects have relatively little effect on our results, and the high energy flow in logged forests is driven primarily by the direct consequences of the logging. For birds the consumption energetics were even stronger in the more heavily logged forests, but this was not the case for the mammals.

Potential effects of temperature variation

All our sites are all in a very similar tropical climate, and hence the major factor driving temperature differences between sites will be microclimatic effects caused by changes in forest structure. Previous studies in the same landscape show that these effects are modest, with the logged forests having peak daytime ambient temperatures around 1 °C

warmer than the old growth forest, and 24-hour mean temperature difference being less that 0.5 °C [4].

For endothermic birds and mammals in warm climates, the sensitivity of metabolism to temperature appears to be negligible or very modest, with body temperatures maintained in the range 36-40°C [5]. A recent review [6] finds that the relationships between the different components of wild mammal and bird daily energy budgets and environmental factors, such as ambient temperature, do not follow universal rules.

As a sensitivity test, if we assume a Q_{10} of 2.0 for Daily Energy Expenditure (*i.e.*, a doubling of metabolic rate for a 10 °C warming) for the observed peak 1°C difference in local microclimate between old growth and logged forests, we would expect the bird and animal metabolism to be 7% higher in the logged forest than if there were no microclimate difference. For a 24-hour mean temperature difference of 0.5 °C, the increase would be only 3.5%. We do not include this calculation given the uncertainties involved, but it shows that any temperature effects are likely to be small, and that our key results are conservative in this respect (*i.e.* the contrast between logged and old growth forests may be slightly greater than we show here if temperature differences were to be taken into account).

Analysis of bird species with particularly mobile life-histories

We used modelled density estimates to generate territory size estimates (assuming non-overlapping territories) and assess the allometric relationship with species' weight. We compared this with the allometric relationships between territory-size estimates and species' average weights for 27 Asian bird species, including seven hornbills we were able to find in the literature. Territory size estimates in the literature were based on alternate approaches including telemetry[7] nest monitoring[8] group-following[9], territory spot-mapping and distance-sampling[10]). The allometric relationships between body mass and territory size

followed [11] and were broadly similar between our modelled estimates and those in the literature. However, our initial density estimates for large-bodied hornbill species were considerable outliers from this pattern. Issues with adequately estimating densities for hornbills based on point count methodologies have been highlighted previously, particularly when encounters of fly-over birds are included [12] as is the case in our dataset. In order to preserve differences in the relative abundance between hornbill species in our landscapes, but control for bias introduced by their different life-history, home-range estimates of each hornbill species in each habitat type were centred around the mean value and scaled to one-unit standard deviation. This was multiplied by a conversion factor of 465.3ha based on the mean home-range reported across the seven species (references above; Supplementary Data Table 1) to calculate scaled home range estimates for each of these species. Per-hectare density estimates were inferred as the inverse of scaled home-range.

Supplementary Discussion References

1. Wearn, O. R. *et al.* Mammalian species abundance across a gradient of tropical land-use intensity: A hierarchical multi-species modelling approach. *Biol. Conserv.* **212**, 162–171 (2017).
2. Struebig, M. J. *et al.* Chapter Three - Quantifying the Biodiversity Value of Repeatedly Logged Rainforests: Gradient and Comparative Approaches from Borneo. in *Advances in Ecological Research* (eds. Woodward, G. & O'Gorman, E. J.) vol. 48 183–224 (Academic Press, 2013).
3. Milodowski, D. T. *et al.* The impact of logging on vertical canopy structure across a gradient of tropical forest degradation intensity in Borneo. *J. Appl. Ecol.* (2021) doi:10.1111/1365-2664.13895.

4. Hardwick, S. R. *et al.* The relationship between leaf area index and microclimate in tropical forest and oil palm plantation: Forest disturbance drives changes in microclimate. *Agric. For. Meteorol.* **201**, 187–195 (2015).
5. Brown, J. H., Gillooly, J. F., Allen, A. P., Savage, V. M. & West, G. B. TOWARD A METABOLIC THEORY OF ECOLOGY. *Ecology* vol. 85 1771–1789 Preprint at https://doi.org/10.1890/03-9000 (2004).
6. Portugal, S. J. *et al.* Associations between Resting, Activity, and Daily Metabolic Rate in Free-Living Endotherms: No Universal Rule in Birds and Mammals. *Physiol. Biochem. Zool.* **89**, 251–261 (2016).
7. Poonswad, P. & Tsuji, A. Ranges of males of the Great Hornbill Buceros bicornis, Brown Hornbill Ptilolaemus tickelli and Wreathed Hornbill Rhyticeros undulatus in Khao Yai National Park …. *Ibis* (1994).
8. Kinnaird, M. F. & O'brien, T. G. Breeding ecology of the Sulawesi Red-Knobbed Hornbill Aceros cassidix. *Ibis* vol. 141 60–69 Preprint at https://doi.org/10.1111/j.1474-919x.1999.tb04263.x (2008).
9. O'brien, T. G. Behavioural ecology of the North Sulawesi Tarictic Hornbill Penelopides exarhatus exarhatus during the breeding season. *Ibis* **139**, 97–101 (2008).
10. Gale, G. A. & Thongaree, S. Density estimates of nine hornbill species in a lowland forest site in southern Thailand. *Bird Conserv. Int.* **16**, 57–69 (2006).
11. Schoener, T. W. Sizes of feeding territories among birds. *Ecology* **49**, 123–141 (1968).
12. Marsden, S. J. Estimation of parrot and hornbill densities using a point count distance sampling method. *Ibis* **141**, 327–390 (1999).

Table S1: Parameter values for the allometric equations for field metabolic rates (FMR) of mammals and birds, derived from [1].

Animal group	*a*	*b*	*Mean log x*	*c*	*d*	*e*	Equation number in [1]
Birds	10.5	0.681	1.950	0.328	1.011	0.012	17: All birds
Mammals	4.21	0.772	2.364	0.423	1.017	0.010	2: Eutherian mammals

FMR = *a** body mass ^ *b*, where the units are kJ day^{-1} for field metabolic rate and g for body mass.

Equation for calculating 95% confidence intervals of the prediction: $95\%CI_{log(FMR)}$= log(predicted FMR) ± c{d + e[log(body mass) – (*mean log x*)]^2}^0.5

Table S2: Assimilation efficiencies (%) used for each feeding group. Uncertainties indicated are standard deviations across studies. Assimilation estimates for birds are by guild; the category 'All birds' is used for those guilds for which data is not available. Derived from [2] for birds and [3] for mammals.

Diet	Invertebrates	Vertebrates	Nuts/seeds	Leaves	Fruit	Nectar
Accipitriformes	82±8.0					
Columbiformes			76±5.4			
Galliformes	70±17	70±17	65±18	42±12	57±20	
Piciformes	64			61		
Passeriformes	76±6.7	76±6.7	80±7.9	76±13.2	67±14.7	90±10.5
All birds	78±9.3	78±9.3	75±14	44±15	63±17	94±11
Mammals	88±5.9	85±5.8	83±8.5	46±10.7 (small mammals) 32±8.4 (large mammals)	83±8.5	-

Table S3. Coefficients of the linear mixed effect model for Energetic Shannon-Wiener index, with Habitat type as fixed effect and Guild (n=18, see Supplementary Data Table 1) as a random effect, with the total number of observations = 54 (Habitat type × Guild combinations).

Habitat type	Value	Standard error [95% confidence intervals]	Degrees of freedom	t-value	p-value
Intercept (Old-growth forest)	1.445	0.242 [0.953, 1.937]	34	5.9681	<0.0001
Logged forest	-0.027	0.075 [-0.180, 0.125]	34	-0.3631	0.7187
Oil palm	-0.711	0.186 [-1.088, -0.333]	34	-3.8264	0.0005

Habitat type is significant, $F_{2,34} = 7.369$, p=0.0022. Standard error of the random intercept = 1.005, with residual standard error = 0.2404.

Supplementary Table References

1. Nagy, K. A., Girard, I. A. & Brown, T. K. Energetics of free-ranging mammals, reptiles, and birds. *Annu. Rev. Nutr.* **19**, 247–277 (1999).
2. Bairlein, F. Energy and nutrient utilization efficiencies in birds--a review. in *Proceedings of the 22nd International Ornithological Congress (ed. NJ Adams and RH Slotow)* 2221–2246 (1999).
3. Crocker, D., Hart, A., Gurney, J., McCoy, C. *Estimating daily food intake of wild birds and mammals*. https://www.hse.gov.uk/pesticides/resources/R/Research_PN0908.pdf (2002).
